# How do new arthroplasty surgeons incorporate technology into their practice?

**DOI:** 10.1007/s11701-026-03385-7

**Published:** 2026-05-04

**Authors:** Laith Bahlouli, Olivia Schaffer, Thomas Bieganowski, Anzar Sarfraz, Farouk Khury, Ran Schwarzkopf, Vinay K. Aggarwal, Joshua C. Rozell

**Affiliations:** 1 Technion – Israel Institute of Technology, The Ruth and Bruce Rappaport Faculty of Medicine, Haifa, Israel; 2 Division of Orthopedic Surgery, Rambam Health Care Campus, Haifa, Israel; 3 Department of Orthopedic Surgery, NYU Langone Health, New York, NY USA

**Keywords:** Adult reconstruction, Survey, Technology, Robot

## Abstract

The use of technology in adult reconstruction (AR) reflects a balance of perceived utility, workflow considerations, and training exposure. This study evaluated whether exposure to technology during residency and fellowship training influences early-career AR surgeons’ utilization of and attitudes towards technology in total joint arthroplasty (TJA). An online survey was distributed to a nationwide cohort of 51 AR surgeons who completed fellowship between 2011 and 2022 at 13 U.S. programs. Survey items assessed exposure to technology during training, utilization, and perceived impact of technology on clinical practice. 36 surgeons (71%) reported using technology in fewer than half their training cases (< 50% group), while 15 (29%) reported use in the majority of cases (> 50% group). Most surgeons (88%) reported access to technology in their current practice, with no statistically significant difference between training exposure groups (*p* = 0.999). Similarly, among those with access, most surgeons (78%) reported using technology in their current practice, with no statistically significant difference between training groups (*p* = 0.238). However, surgeons with greater exposure rated the importance of technology in TJA and its impact on patient outcomes significantly higher (*p* = 0.003 for both). Greater exposure to technology during training was thus associated with higher perceived value, though no significant differences in access or utilization in early practice were observed.

## Introduction

Robotic and computer-assisted technologies are becoming increasingly popular in adult reconstructive (AR) surgery, largely due to perceived improvements in precision and accuracy. Prior survey data show that 73.1% of robotic users identify increased precision as their main motivation [1–3]. Adoption is also driven by workflow considerations, such as surgical efficiency [4, 5]. Nonclinical pressures may contribute to the increase in technology usage, with nearly one in five robotic users reporting that marketing, administrative encouragement, or peer influence factored into their decision to use these systems [1].

Several barriers to adoption influence technology utilization patterns. Increased operative time remains a consistent concern, with 76.5% of surgeons in one survey reporting longer procedures when using robotics [1–3]. A majority of surveyed surgeons do not feel that robotic assistance is cost-effective, and economic analyses show higher overall costs for robotic and computer-navigated procedures compared to conventional arthroplasty [1, 6]. Adoption also comes with a meaningful learning curve, and many surgeons estimate that 20 to 40 cases are needed to gain comfort with robotic workflows [1].

Despite these barriers, utilization has risen substantially over the past several years [4, 5]. Younger surgeons with fewer than ten years in practice report particularly strong interest in these technologies [5]. Rates also vary by procedure: partial knee arthroplasty has the highest rate of robotic use at 43%, followed by total knee arthroplasty (TKA) at 36% and total hip arthroplasty (THA) at 20% [5]. Although technology-assisted techniques reliably improve radiographic alignment and component positioning, most comparative studies show similar patient-reported outcomes and complication rates relative to conventional techniques [2, 3, 6–14].

Training exposure demonstrates considerable variability across institutions. While 68% of residents report access to technology-assisted arthroplasty at their training sites, only a minority feel comfortable using technology or receive structured hands-on training [15–17]. Geographic and institutional differences further shape access due to the higher concentration of robotic arthroplasty in specific regions and at high-volume centers [18, 19]. Many trainees also express concern that excessive reliance on robotics can limit skill development with conventional instrumentation, and most residents believe that traditional techniques should be mastered before incorporating technology [15, 16]. Fellowship applicants now generally place relatively low importance on robotic exposure when evaluating adult reconstruction programs [20].

Technology adoption in adult reconstruction reflects a complex balance of perceived accuracy gains, workflow considerations, costs, time demands, institutional availability, and variable training exposure. The purpose of this study is to determine whether exposure to technology during training influences early-career adult reconstruction surgeons’ opinions about and utilization of technology during total joint arthroplasty. These insights can help inform future fellowship training curricula and guide the design and implementation of technologies that align with the needs of arthroplasty surgeons.

## Materials and methods

### Survey characteristics

A total of 51 orthopaedic AR surgeons responded to a 15-question electronic survey. The survey was distributed to AR fellowship program directors, who were asked to disseminate it to their program graduates. Respondents completed fellowship between 2011 and 2022, representing 13 fellowship programs across the United States. The survey was sent via REDCap (Project REDCap, Nashville, Tennessee), and data were collected using Microsoft Excel (Microsoft, Redmond, Washington). Participation was voluntary, and responses were recorded anonymously.

Survey questions are provided in the Appendix. The initial inquiries queried surgeons about their current practice setting, years in practice following AR fellowship, annual total joint arthroplasty (TJA) volume, any technologies they have experience with, and the percentage of cases during residency and fellowship training that involved technology. Surgeons were subsequently stratified into two groups based on training exposure to technology: those who reported technology use in less than half of residency and fellowship cases (< 50% group) and those who reported use in more than half of cases (> 50% group). The second set of inquiries assessed current access to technology in their practice. If surgeons reported access, they were asked how long technology had been available in their practice, which specific technologies they had access to, whether they used technology during their arthroplasty procedures, and why they chose to use certain technologies over others. Surgeons without access to technology were asked about any barriers to implementation. The final section surveyed opinions regarding technology in AR practice and used a five-point Likert scale, where 1 indicated “not important” and 5 indicated “extremely important.”

## Statistical analyses

Statistical analyses were performed using R version 4.4.1 (R Foundation for Statistical Computing, Vienna, Austria). Categorical variables were summarized as counts and percentages, and compared using Fisher’s exact test. Continuous and ordinal variables were summarized as means and standard deviations, and compared using the Wilcoxon rank-sum test. A *p*-value < 0.05 was considered statistically significant.

## Results

A total of 51 surgeons completed the survey. Technology was used in < 50% of training cases for 71% of surgeons, while 29% reported technology use in > 50% of training cases. Most surgeons practiced in private settings (41%), followed by academic/university institutions (33%), hospital-employed practices (28%), and government or Veterans’ Affairs (VA) institutions (2%). There were no statistically significant differences in practice settings between the < 50% and > 50% training groups (private: *p* = 0.999; academic: *p* = 0.101; hospital: *p* = 0.513; government: *p* = 0.999). The mean duration in practice following AR fellowship was 6.0 ± 6.1 years and did not differ significantly between training groups (*p* = 0.349). Most surgeons (73%) performed > 200 TJAs annually, with no statistically significant difference between training groups (*p* = 0.999) (Table 1).

**Table 1:** Baseline characteristics across training groups

Question, *n* (%)	All *n* = 51	< 50% Tech *n* = 36	> 50% Tech *n* = 15	*P*-value
In what type of practice setting do you currently perform most of your total joint procedures?*				
Academic Government Hospital-Employed Private	17 (33.3) 1 (2.0) 14 (27.5) 21 (41.2)	9 (25.0) 1 (2.8) 11 (30.6) 15 (41.7)	8 (53.3) 0 (0.0) 3 (20.0) 6 (40.0)	0.101 0.999 0.513 0.999
**How many years have you been in practice following AR fellowship?** mean [SD]	6.0 [6.1]	6.3 [6.3]	5.2 [5.5]	0.349
**Approximately how many total joint arthroplasties (hip and knee) do you perform each year?**				
<200 >200	14 (27.5) 37 (72.5)	10 (27.8) 26 (72.2)	4 (26.7) 11 (73.3)	0.999
Which forms of technology do you currently have experience with?*				
Accelerometer Navigation OrthoSensor Patient-Specific Instrumentation Robot	21 (41.2) 40 (78.4) 17 (33.3) 23 (45.1) 42 (82.4)	15 (41.7) 26 (72.2) 11 (30.6) 16 (44.4) 31 (86.1)	6 (40.0) 14 (93.3) 6 (40.0) 7 (46.7) 11 (73.3)	0.999 0.141 0.532 0.999 0.421
**Do you currently have access to robotics or navigation in your practice?**				
Yes No	45 (88.2) 6 (11.8)	32 (88.9) 4 (11.1)	13 (86.7) 2 (13.3)	0.999

n, number of; %, percent; SD, standard deviation; AR, adult reconstruction. <50% Tech and > 50% Tech indicate the proportion of training cases performed using technology. **Bold** indicates statistical significance (*p* < 0.05)

*Respondents could pick more than one option

Regarding specific technologies, 82% of surgeons had experience with robotic assistance, 78% with navigation, 45% with patient-specific instrumentation, 41% with accelerometers, and 33% with OrthoSensor. There were no statistically significant differences in experience with any form of technology between training exposure groups (robot: *p* = 0.421; navigation: *p* = 0.141; patient-specific: *p* = 0.999; accelerometer: *p* = 0.999; OrthoSensor: *p* = 0.532). Additionally, 88% of respondents reported having access to technology in their practice, which did not differ significantly between training groups (*p* = 0.999) (Table 1).

Among surgeons with access to technology (*n* = 45), 69% had access for less than five years, with no statistically significant difference between training groups (*p* = 0.787). Access to specific technologies among these surgeons was as follows: robotics (89%), navigation (60%), patient-specific instrumentation (44%), accelerometer (33%), and OrthoSensor (20%). None of these differed significantly between training groups (robot: *p* = 0.301; navigation: *p* = 0.514; patient-specific: *p* = 0.515; accelerometer: *p* = 0.999; OrthoSensor: *p* = 0.094). The majority of surgeons with access to technology reported using it during TJA (78%), which was not significantly different between training groups (*p* = 0.238) (Table 2).

**Table 2:** Technology usage among surgeons with access to robotics or navigation, across training groups

Question, *n* (%)	All *n* = 45	< 50% Tech *n* = 32	> 50% Tech *n* = 13	*P*-value
**How many years have these technologies been available in your practice?**				
<5 Years >5 Years	31 (68.9) 14 (31.1)	20 (62.5) 12 (37.5)	11 (84.6) 2 (15.4)	0.787
Which forms of technology do you currently have access to?*				
Accelerometer Navigation OrthoSensor Patient-Specific Instrumentation Robot	15 (33.3) 27 (60.0) 9 (20.0) 20 (44.4) 40 (88.9)	11 (34.4) 18 (56.3) 4 (12.5) 13 (40.6) 27 (84.4)	4 (30.8) 9 (69.2) 5 (38.5) 7 (53.8) 13 (100.0)	0.999 0.514 0.094 0.515 0.301
**Do you use any form of technology during your total joint procedures?**				
Yes No	35 (77.8) 10 (22.2)	23 (71.9) 9 (28.1)	12 (92.3) 1 (7.7)	0.238
If yes to above, why have you chosen to use certain forms of technology over others*				
Administrative Recommendation Colleague Recommendation Manufacturer-Provided Technology Marketing Purposes	4 (11.4) 10 (28.6) 13 (37.1) 3 (8.6)	3 (13.0) 5 (21.7) 9 (39.1) 3 (13.0)	1 (8.3) 5 (41.7) 4 (33.3) 0 (0.0)	0.999 0.258 0.999 0.536

n, number of; %, percent. <50% Tech and > 50% Tech indicate the proportion of training cases performed using technology. **Bold** indicates statistical significance (*p* < 0.05)

*Respondents could pick more than one option

Among surgeons who used technology (*n* = 35), when queried for their reasoning for selecting certain forms of technology, 37% reported manufacturer-provided technology as a reason for use, 29% cited colleague recommendation, 11% reported hospital administration recommendation, and 9% cited marketing purposes. This reasoning did not differ significantly between training groups (manufacturer: *p* = 0.999, colleague: *p* = 0.258; administrative: *p* = 0.999, marketing: *p* = 0.536) (Table 2).

When asked about the importance of exposure to technology during training, the mean Likert score of the 51 surgeons was 3.9 ± 1.0, with no statistically significant difference between training groups (*p* = 0.140). For the importance of technology use during TJA, the mean score was 2.7 ± 1.4. Surgeons with > 50% of training cases involving technology had significantly higher scores (< 50%: 2.3 ± 1.2; >50%: 3.6 ± 1.2; *p* = 0.003). Regarding the importance of technology in improving postoperative outcomes, the mean score was 2.6 ± 1.3, and was also significantly higher among surgeons with > 50% training case technology exposure (< 50%: 2.3 ± 1.2; >50%: 3.5 ± 1.2; *p* = 0.003) (Table 3).

**Table 3:** Opinions of technology across training groups

Question, *n* (%)	All *n* = 51	< 50% Tech *n* = 36	> 50% Tech *n* = 15	*P*-value
**How important is exposure to technology during residency and fellowship training?**				
Not important [1] Somewhat unimportant [2] Neutral [3] Somewhat important [4] Extremely important [5] Mean [SD]	2 (3.9) 1 (2.0) 10 (19.6) 24 (47.1) 14 (27.5) 3.9 [1.0]	2 (5.6) 0 (0.0) 9 (25.0) 17 (47.2) 8 (22.2) 3.8 [1.0]	0 (0.0) 1 (6.7) 1 (6.7) 7 (46.7) 6 (40.0) 4.2 [0.9]	0.140
**How important is the use of technology during your total joint arthroplasty procedures?**				
Not important [1] Somewhat unimportant [2] Neutral [3] Somewhat important [4] Extremely important [5] Mean [SD]	13 (25.5) 10 (19.6) 13 (25.5) 9 (17.6) 6 (11.8) 2.7 [1.4]	12 (33.3) 8 (22.2) 10 (27.8) 4 (11.1) 2 (5.6) 2.3 [1.2]	1 (6.7) 2 (13.3) 3 (20.0) 5 (33.3) 4 (26.7) 3.6 [1.2]	**0.003**
**How important is technology to the improvement of patient outcomes after surgery compared to conventional methods?**				
Not important [1] Somewhat unimportant [2] Neutral [3] Somewhat important [4] Extremely important [5] Mean [SD]	14 (27.5) 10 (19.6) 13 (25.5) 10 (19.6) 4 (7.8) 2.6 [1.3]	13 (36.1) 8 (22.2) 9 (25.0) 5 (13.9) 1 (2.8) 2.3 [1.2]	1 (6.7) 2 (13.3) 4 (26.7) 5 (33.3) 3 (20.0) 3.5 [1.2]	**0.003**

n, number of; %, percent; SD, standard deviation. <50% Tech and > 50% Tech indicate the proportion of training cases performed using technology. **Bold** indicates statistical significance (*p* < 0.05)

Of the 51 surgeons, six (12%) reported not having access to technology in their practice. When queried about any barriers to incorporating technology that they had experienced, all indicated either satisfactory outcomes without technology or insufficient evidence supporting its superiority over conventional methods. Of these six surgeons, three used technology in < 10% of training cases, one had no exposure to technology or it was unavailable during training, and two had > 50% training case exposure. Their mean Likert score was 4.2 ± 0.8 for the importance of exposure during training, 1.8 ± 1.2 for the importance of technology use during procedures, and 2.8 ± 1.2 for the importance of technology in improving patient outcomes.

## Discussion

Nearly three-quarters of our respondents affirmed that exposure to technology during residency and fellowship training is important, with no statistically significant difference in mean Likert score between training exposure groups. Given that our cohort largely represents early-career surgeons, with a mean of six years in practice following AR fellowship, these findings reflect a growing interest in technology among younger surgeons. Supporting this trend, Tamer et al. found that interest in robotics increased from 30% to around 70% between 2018 and 2023 for surgeons less than ten years in practice [5]. Similarly, in a national survey of 280 orthopaedic residents and fellows, Leroy et al. found that over 60% of respondents believed that robotics represents an exciting future and area for advancement in orthopaedics, 90% felt it is here to stay, and more than 45% expressed interest in incorporating robotics into their future practices [17].

Prior work has also suggested a potential educational value of technology during training. Deckey et al. proposed that robotic technology may serve as an effective learning tool by providing visual preoperative planning and real-time intraoperative feedback, enabling evaluation of component alignment and soft-tissue balancing [21]. Consistent with this, 71.4% of respondents in the study by Leroy et al. believed that robotics could facilitate resident education through its preoperative planning and simulation of implant placement [17]. Additionally, in a survey of 220 senior orthopaedic residents, Duensing et al. reported that approximately one-third felt robotic assistance in knee arthroplasty improved their understanding of the procedure [16]. Collectively, these findings suggest that although many of our respondents did not use technology in the majority of their training cases, interest in technology remains high among early-career orthopaedic surgeons, potentially reflecting its perceived clinical and educational value, as well as its reliability and consistency.

Although the majority of respondents reported access to and use of technology in their current practices, more than 70% reported using technology in fewer than half of their training cases. This discrepancy highlights a mismatch between the widespread adoption of technology in contemporary practice and the extent of formal exposure during training. Leroy et al. found that over two-thirds of their respondents did not feel they had received adequate robotic training, and more than 44% reported lacking autonomy to use technology during training cases [17]. Additionally, nearly half of respondents reported not having a mentor excited about robotics [17]. Similarly, in the survey administered by Duensing et al., 37% of respondents indicated that they wished their training program offered greater exposure to robotic knee replacement [16].

Although the underlying causes for these perceived training gaps were not directly examined, findings from a national survey of 735 members of the American Association of Hip and Knee Surgeons (AAHKS) by Sherman and Wu suggest that workflow-related factors may contribute [1]. In their study, 76.5% of surgeons reported longer procedural times when using robotics, and 81.8% indicated that over 20 cases were required to achieve comfort with robotic workflows [1]. Collectively, these factors may limit opportunities for hands-on technology experience and teaching on busy operative days, particularly in settings where trainees have limited autonomy. As the use of technology in TJA continues to expand, its integration into surgical training warrants scrutiny to ensure adequate experiential learning for trainees.

Our survey did not detect a statistically significant difference between surgeons with high and low technology training volume regarding current access to robotics or navigation (*p* = 0.999) or use of technology (*p* = 0.238). In contrast, LeRoy et al. reported that orthopaedic residents and fellows who had robotics training were significantly more likely to express an intention to use robotics in future practice (52.5% vs. 40.0%, *p* = 0.04) [17]. Similarly, Sherman and Wu found that prior training exposure to robotic technique was more prevalent among surgeons who used technology compared with those who did not (73.7% vs. 54.2%, *p* < 0.001) [1]. These differences in our findings likely reflect an important methodological distinction in how training exposure was defined across studies. While LeRoy et al. and Sherman and Wu stratified by binary training exposure to robotics specifically (exposed vs. not exposed), our cohort was stratified by relative training volume involving any form of technology (< 50% of training cases vs. >50% of training cases), with only two surgeons reporting no exposure during training [1, 17]. As a result, our analysis may have captured more nuanced but less polarized differences in exposure, potentially attenuating observed associations with access or use.

Our study also found that surgeons who used technology in the majority of their training cases rated both the importance of technology during TJA and its importance in improving patient outcomes significantly higher than those with less training exposure (*p* = 0.003 for both). While no prior studies have directly examined the relationship between training exposure and perceived value of technology, Sherman et al. reported that surgeons with a conflict of interest (consultancy or financial interest in robotics) were more likely to endorse that robotic arm assistance led to superior long-term functional outcomes, fewer revisions, and fewer complications [1]. Notably, surgeons with and without such conflicts did not differ in their training exposure, suggesting that perceptions of technology may be shaped by experiential or contextual factors beyond formal training alone [1]. Taken together, these findings suggest that while training exposure may not independently determine technology adoption, it may contribute to how surgeons perceive the value of emerging technologies within clinical practice.

There are several potential limitations to this study. The relatively small sample size limits the statistical power of the study and increases the risk of Type II error. As such, non-significant findings should be interpreted with caution, as differences between groups may not have been detected. Additionally, because the survey was distributed through fellowship program directors to their graduates, the total number of recipients is unknown, and a response rate could not be determined. Furthermore, grouping multiple forms of technology into a single training exposure variable may obscure differences between trainees exposed to specific modalities, such as robotics or navigation, and reflects a limitation of the study design. Although the survey was developed by fellowship-trained arthroplasty surgeons, the absence of a validated instrument for assessing experience with and attitudes towards technology in arthroplasty may have introduced response bias related to question phrasing. Importantly, the survey included only surgeons who graduated between 2011 and 2022, which may limit the generalizability of these findings to surgeons with longer time in practice or to those who have more recently completed training. This may also introduce variability in exposure to, use of, and perceptions of technology across training eras. However, respondents represented graduated from 13 different fellowships across the United States, which may enhance the applicability of these findings to early-career arthroplasty surgeons.

## Conclusion

Although limited exposure to technology during training did not restrict access or use in early practice, greater exposure was associated with a higher perceived value of technology. These findings suggest that training experience may play an important role in shaping perceptions of emerging technologies and should be considered in the development of future fellowship curricula and technology implementation strategies within the arthroplasty community.

## Data Availability

No datasets were generated or analysed during the current study.

## Appendix

**1. In what zip code do you currently perform most of your procedures?**

*Free Text*.

**2. In what type of practice setting do you currently perform most of your total joint procedures?**

☐ Academic (University) ☐ Hospital Employed ☐ Private.

☐ Government (Military/VA).

**3. How many years have you been in practice following adult reconstruction (AR) fellowship?**

*Free Text*.

**4. Approximately how many total joint arthroplasties (hip and knee) do you perform each year?**

☐ <50 ☐ 50–100 ☐ 101–150 ☐ 151–200 ☐ >200.

**5. How much of your residency and fellowship training involved experience with any form of technology?**

☐ Almost all cases.

☐ Most cases (> 50%).

☐ Some cases (< 50%).

☐ Very few cases (< 10%).

☐ None/not available during my training.

**6. Which forms of technology do you currently have experience with?**

☐ Navigation.

☐ Orthosensor.

☐ Patient Specific Instrumentation.

☐ Robot.

☐ Accelerometer.

**7. Do you currently have access to robotics or navigation in your practice?**

☐ Robotics ☐ Navigation ☐ Both ☐ None.

*If you answered* *robotics*, *navigation*, *or* *both* *to question #7*:

**8. How many years have these technologies been available in your practice?**

☐ *≤* 1 ☐ 2–5 ☐ 6–10 ☐ 10–20 ☐ >20.

*If you answered* *robotics*, *navigation*, *or* *both* *to question #7*:

**9. Which forms of technology do you currently have access to?**

☐ Navigation.

☐ Orthosensor.

☐ Patient Specific Instrumentation.

☐ Robot.

☐ Accelerometer.

*If you answered* *robotics*, *navigation*, *or* *both* *to question #7*:

**10. Do you use any form of technology during your total joint procedures (select all that apply)?**

☐ Yes, during Total Knee Arthroplasty (TKA).

☐ Yes, during Total Hip Arthroplasty (THA).

☐ Yes, during revision TKA.

☐ Yes, during revision THA.

☐ No, I never use robotics or navigation.

*If you answered* *yes* *to question #10*:

**11. Why have you chosen to use certain forms of technology over others (select all that apply)?**

☐ Anecdotal evidence by colleagues who advocate potentially beneficial outcomes.

☐ The technology I use is marketed and provided by the implant manufacturer that I primarily use.

☐ The technology I use is mostly for marketing purposes.

☐ The technology I use is recommended to me by administrative representatives at my hospital.

☐ Something else (please specify): ______________.

*If you answered* *none* *to question 7*:

**12. What barriers do you face to incorporating technology into your practice (select all that apply)?**

☐ I feel my patient outcomes and my practice are doing well enough without the implementation of technology.

☐ I am not familiar enough with the various systems to implement them.

☐ Lack of evidence demonstrating the superiority of technology over conventional methods.

☐ Lack of availability of different machines and/or continuing training within my practice.

☐ Something else (please specify): ________________.

### Please rate questions 13–15 based on your own opinion using the following scale:

1 – Not important, 2 – somewhat unimportant, 3 – neutral, 4 – somewhat important, 5 – Extremely important.

**13. How important is exposure to technology during residency and fellowship training?**

1 2 3 4 5.

**14. How important is the use of technology during your total joint arthroplasty procedures?**

1 2 3 4 5.

**15. How important is technology to the improvement of patient outcomes after surgery compared to conventional methods?**

1 2 3 4 5.
